# Sense of coherence and diabetes: A prospective occupational cohort study

**DOI:** 10.1186/1471-2458-8-46

**Published:** 2008-02-06

**Authors:** Anne M Kouvonen, Ari Väänänen, Stephen A Woods, Tarja Heponiemi, Aki Koskinen, Salla Toppinen-Tanner

**Affiliations:** 1 Institute of Work, Health & Organisations, University of Nottingham, 8 William Lee Buildings, Nottingham Science and Technology Park, University Boulevard, Nottingham NG7 2RQ, UK; 2 Finnish Institute of Occupational Health, Topeliuksenkatu 42 a A, FIN-00250 Helsinki, Finland; 3 National Research and Development Centre for Welfare and Health (STAKES), POB 220, FIN-00531 Helsinki, Finland; 4 Department of Psychology, University of Helsinki, POB 9, FIN-00014 University of Helsinki, Finland

## Abstract

**Background:**

Sense of coherence (SOC) is an individual characteristic related to a positive life orientation leading to effective coping. A weak SOC has been associated with indicators of general morbidity and mortality. However, the relationship between SOC and diabetes has not been studied in prospective design. The present study prospectively examined the relationship between a weak SOC and the incidence of diabetes.

**Methods:**

The relationship between a weak SOC and the incidence of diabetes was investigated among 5827 Finnish male employees aged 18–65 at baseline (1986). SOC was measured by questionnaire survey at baseline. Data on prescription diabetes drugs from 1987 to 2004 were obtained from the Drug Imbursement Register held by the Social Insurance Institution.

**Results:**

During the follow-up, 313 cases of diabetes were recorded. A weak SOC was associated with a 46% higher risk of diabetes in participants who had been =<50 years of age on entry into the study. This association was independent of age, education, marital status, psychological distress, self-rated health, smoking status, binge drinking and physical activity. No similar association was observed in older employees.

**Conclusion:**

The results suggest that besides focusing on well-known risk factors for diabetes, strengthening SOC in employees of =<50 years of age can also play a role in attempts to tackle increasing rates of diabetes.

## Background

The prevalence of diabetes is rapidly increasing. This is the case especially for Type-2 diabetes [1]. According to the Global Burden of Disease Study by the World Health Organization (WHO), the total number of people with diabetes is estimated to rise from 171 million (2.8%) in 2000 to 366 million (4.4%) in 2030 and this figure is probably an underestimate [2]. The National Health and Nutrition Examination Survey (NHANES III) reports that in the US population over 65 years of age, 18% to 20% have diabetes, with 40% having either diabetes or its precursor form of impaired glucose tolerance [3].

Diabetes is becoming more prevalent also in Finland. In 1991, 100,000 people used prescription diabetes drugs reimbursable under the National Insurance scheme. By 2004 this figure rose to 161,305. It was estimated that in 2004 about 200,000 Finns suffered from diabetes (4% of the population). Of these, the vast majority, 190,000 had Type-2 diabetes. Similarly to other countries, diabetes was more common among men, older and less educated people [4]. Type-2 diabetes is still rather rare in under 51-year-old Finns [5].

Given that Type-2 diabetes is partly preventable, it is important to identify not only physical and health behavioural risk factors but also psychological factors that can contribute to promoting good health [6]. The concept of sense of coherence (SOC) was introduced by Antonovsky as a part of the salutogenic theory, which examines the question why some people regardless of encountering stressors and major life events stay healthy while others do not [7]. SOC comprises three dimensions, that is, comprehensibility, manageability, and meaningfulness. Comprehensibility concerns the tendency to perceive stimuli in a clear, ordered and structured way. Manageability concerns a generalised perception about the adequacy and sufficiency of coping resources available to the individual. Meaningfulness refers to the extent that a person believes that the investment of activity and energy in their life tasks is worthwhile, thereby reflecting affectivity and motivation. SOC can be viewed as an enduring person or 'view-of-life' characteristic that stabilises in early adulthood [8]. According to Antonovsky [7], individuals with a strong SOC are more capable of perceiving stressors with sense and structure, and are more efficacious about their ability to deal with them.

Furthermore, in the SOC theory it is hypothesised that SOC influences an individual's position on the health – disease continuum. Therefore, in a given situation two individuals with a weak SOC might, after encountering stressors, end up with different outcomes on their health much depending on their personal, possibly bio-medically determined qualities.

A recent systematic review on Antonovsky's SOC scale and health identified SOC as a health promoting resource, which strengthens resilience and develops a positive subjective state of health [9]. Even if the theory of SOC is oriented towards causes of health rather than illness, in the literature negative health outcomes of weak SOC have been widely studied. A weak SOC has predicted psychological strain [10]. In longitudinal studies, a weak SOC has also been associated with various indicators of poor health, such as an increased incidence of medically certified sickness absence [11], an increased risk of myocardial infarction [12] and an increased all cause mortality [13].

Although many factors related to a weak SOC, such as inadequate coping skills, higher chronic strain and health-damaging behaviours are likely to be associated with an increased risk of diabetes, little is known about whether a weak SOC elevates the risk of diabetes. It can be hypothesized, however, that people with a weak SOC have an increased risk of diabetes because of increased vulnerability to environmental stressors and ineffective responses to them. In previous studies, a weak SOC has been associated with health-risk behaviours, such as hazardous alcohol consumption [14], lower physical activity [15] and unhealthy food choices [16]. Two recent Swedish cross-sectional studies addressed the relationship between SOC and Type-2 diabetes [17,18]. In both of these studies, a significant association was found between a weak SOC and diabetes. However, as reversed causality can explain these associations, prospective studies are needed.

The importance of SOC in the development of chronic disease may depend on the age of the person. Recently it was found that weak SOC increased the likelihood of a grant of disability pension among employees who were =<50 years of age at baseline [19].

Our prospective cohort study investigated whether differences in SOC predicted incidence of diabetes up to 18 years later in initially healthy male employees. In our study, we controlled for several baseline factors associated with both SOC and diabetes. Employees who were =<50 years of age and older employees were examined separately. Given the long-term development of impaired glucose tolerance and the high average age of onset of diabetes [5], we assume that in 18–50-year-olds the long-term preventive power of SOC on diabetes could be particularly observed, whereas in older industrial employees poor health and other bio-medical risk factors related to diabetes are more likely to explain the onset of diabetes.

## Methods

This study is part of the ongoing Still Working -Study examining a wide range of long-term antecedents of health, morbidity and mortality in a 18-year follow-up among industrial white- and blue-collar employees. The survey data are initially based on a long-term research programme of the Finnish Institute of Occupational Health within a multinational forest industry corporation originally founded in Finland [20].

**Figure 1:**
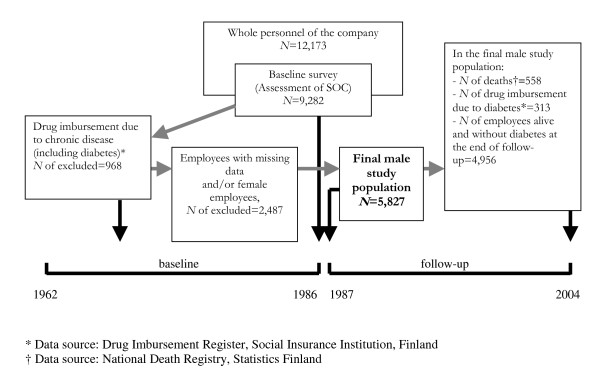
Study design, sample selection, and description of the final study population.

Study design, sample selection, and description of the final study population are presented in Figure 1. In 1986, a questionnaire survey on demographics, psychosocial characteristics and health-risk behaviours was sent to all 12,173 (69% blue-collar workers, 77% male) employees of this company in Finland. Altogether 76% of the personnel responded to the questionnaire, the lowest response rate being among the hourly-paid blue-collar workers (69%).

We included only male employees in our study. The prevalence of diabetes is generally higher among men than among women [4,21]. In our study population, the number of diabetic women was very low in both age groups (*N *= 47 in the women less than 51 years of age and *N *= 14 in the women over 50 years of age) (data not shown). Hence, the analyses among the subgroup of female employees are likely to produce results with poor validity.

Those men who were up to the survey year 1986 (including 1986) free from diabetes and other chronic diseases, who responded to the scale of sense of coherence and other survey items under the study, and who were identified from the database of the National Population Register Centre were included in the final cohort of 5827 employees. At baseline, their mean age was 39.4 years (standard deviation 10.4; range 18–65) and the average organisational tenure was 15.6 years (range 1–45). Of them, 4192 were blue-collar employees who typically worked as monitors of the machines in the industrial plants and maintenance occupations while the white-collar employees (*N *= 1635) were mostly employed as managers, foremen, and technical staff. Other baseline characteristics are presented in Table 1.

**Table 1:** Mean of sense of coherence (SOC), age adjusted hazard ratios (HRs) and their 95 percent confidence intervals (95% CIs) for drug imbursement due to diabetes during the follow-up of 18 years by confounding variables at baseline in initially healthy male employees

**Characteristic**	***N***	**Mean of SOC (95% CI)**	***p*-value**	**No. of diabetes cases**	**HR (95% CI)**
Age at baseline (years)			<0.001		
=<50	5058	64.30 (64.00–64.59)		254	1.00
>50	769	65.90 (65.13–66.65)		59	1.73 (1.30–2.29)
Education			<0.001		
Tertiary	806	67.05 (66.26–67.79)		40	1.00
Secondary	2728	64.46 (64.05–64.87)		119	0.91 (0.63–1.30)
Primary	2293	63.67 (63.22–64.11)		154	1.39 (0.98–1.97)
Marital status			<0.001		
Married	4123	65.28 (64.95–65.62)		226	1.00
Not married	1704	62.62 (62.09–63.15)		87	0.99 (0.77–1.28)
Psychological distress			<0.001		
Low	4437	67.36 (67.08–67.64)		238	1.00
High	1390	55.39 (54.89–55.89)		75	1.01 (0.78–1.31)
Self-rated health			<0.001		
Good	5516	64.85 (64.57–65.13)		282	1.00
Poor	311	58.36 (57.17–59.55)		31	1.98 (1.36–2.87)
Smoking status			<0.001		
No	3696	64.77 (64.43–65.12)		183	1.00
Yes	2131	64.04 (63.58–64.50)		130	1.37 (1.09–1.71)
Binge drinking*			<0.001		
No	4881	65.19 (64.90–65.49)		242	1.00
Yes	946	60.95 (60.27–61.63)		71	1.67 (1.28–2.18)
Physical activity †			<0.001		
High or moderate	3608	65.10 (64.75–65.45)		165	1.00
Low	2219	63.54 (63.09–63.99)		148	1.52 (1.22–1.90)

* Frequency of binge drinking: No = excessive drinking leading to intoxication less than twice per month; Yes = excessive drinking leading to intoxication twice or more per month

† Frequency of exercising: High or moderate = Once a week or more; Low = Less than once a week

Information on entitlement to drug imbursement due to diabetes during the period between January 1, 1987 and December 31, 2004 was derived from the national register held by the Social Insurance Institution, Finland, and linked to the data by means of each participant's id number. Id number is a unique number that all Finnish citizens are given at birth and which is used for all contacts with welfare and health care organisations. All study phases were approved by the ethics committee of the Finnish Institute of Occupational Health.

### Measures

#### Sense of coherence

SOC was assessed with a 13-item version of Antonovsky's Orientation to Life Questionnaire measuring the three aspects of SOC, that is, meaningfulness, comprehensibility, and manageability [7]. The respondents were asked to check their level of agreement with each of the items on a seven-point scale (Cronbach's alpha = 0.84). The example items are as follows: "Most of the things you do in the future will probably be completely fascinating" (an item assessing meaningfulness)"; "Do you have the feeling that you are in an unfamiliar situation and don't know what to do?" (comprehensibility); and "How often do you have the feeling that you're not sure you can keep things under control?" (manageability) [7]. This summary score has been used in various studies on Finnish employees and the validity of the scale has been found to be good [11,22-24].

A summary score of ratings of all SOC items was constructed by adding up the scores of individual items [22]. In our sample, the mean of SOC was 64.51 (standard deviation (SD) 10.81). A high score in the scale indicates a strong SOC. For the analysis, the summary score was divided into tertiles indicating weak, medium and strong SOC.

#### Drug imbursement due to diabetes

We collected data on drugs reimbursable under the National Insurance scheme. All persons who were eligible for reimbursement of medicine due to diabetes (yes vs. no) or some other severe disease (yes vs. no) before the assessment of SOC (1964–1986), during the assessment of SOC (in 1986), and after the assessments were obtained from the Drug Imbursement Register held by the Social Insurance Institution, Finland. This national registry covers virtually all information on the entitlement to reimbursed drugs relating to long-term chronic illnesses for each Finnish citizen residing in Finland, regardless of age, sex and educational attainment. The register of the Social Insurance Institution is comprised of outpatient data based on the WHO Anatomical Therapeutic Chemical (ATC) classification [25]. All entitlements for drug imbursement are prescribed by a physician and authorized by the Social Insurance Institution. The first date of the right for the reimbursed drug due to diabetes was obtained for all the study participants and non-participants, who were initially free from diabetes and other chronic diseases according to the Drug Imbursement Register.

#### Ascertainment of mortality

Mortality data from 1 April 1986 to 31 December 2004 were obtained from the National Death Registry kept by Statistics Finland (official Finnish government statistics). The database provides virtually complete population mortality data [26]. The dates and causes (from death certificates) of death were obtained for all the participants.

#### Other variables

Data on age and marital status (married vs. not married) were obtained from the National Population Register Centre, while educational attainment was assessed with the questionnaire. The categories of educational attainment were as follows: basic education (primary school), secondary education, and tertiary education (institute, college or university).

The following health-risk behaviours were measured at baseline using the questionnaire: regular smoking (yes vs. no), binge drinking (excessive drinking leading intoxication twice or more per month vs. less than twice per month) [27], and physical activity. The response options for physical activity (frequency of exercise) were as follows: 1 = 'daily or nearly daily', 2 = 'once a week', 3 = 'two times a month', 4 = 'a few times a year', 5 = 'never'. As regular physical exercise at least once a week has been associated with a reduced risk of type 2 diabetes in working age men [28], this variable was dichotomised for the analysis (exercising once a week or more vs. less than once a week).

It is possible that psychological distress and poor subjective health can indicate sub-clinical diabetes and predict the subsequent incidence of diabetes. Therefore they were controlled for in this study. Psychological distress was assessed in the survey with an 11-item four-point scale measuring insomnia, depressive symptoms, tension and anxiety [29,30]. A summary scale was formed and dichotomised (the respondents in the highest quartile were defined as having high distress, Cronbach's alpha = 0.89). Poor self-rated health was indicated by health ratings less than good on a five-point single item scale: "How would you estimate your current state of health compared to your age mates?"[31]. The response alternatives were as follows: 1 = very poor, 2 = poor, 3 = average, 4 = good, 5 = very good. The measure was dichotomized by grouping the response scores 1 to 3 into the category of poor self-rated health and scores 4 and 5 into the category of good self-rated health.

### Statistical Analysis

Means and 95% confidence intervals (CIs) of SOC were first computed for each diabetes risk factor. Significance of differences in distribution of SOC in relation to each of the potential confounding variables was determined by means of analysis of variance. Associations between SOC and diabetes were assessed with Cox proportional-hazards models. For each participant, person-days of follow-up were calculated from January 1, 1987 to the death, to the entitlement to drugs related to diabetes or to December 31, 2004, whichever of these three options came first. The time-dependent interaction term between predictor and logarithm of follow-up period was nonsignificant, confirming that the proportional hazards assumption was justified (*p = *0.42). Hazard ratios (HRs) and 95% CIs for tertiles of SOC (weak, medium, strong) provided risk estimates. At the first stage, age-adjusted HRs and 95% CIs for new entitlement for diabetes medication were calculated by potential confounding variables at baseline (Table 1).

At the second stage, the SOC variable was regressed together with baseline age, educational attainment, marital status, psychological distress and self-rated health (Table 2, Model 1). At the third stage, the SOC variable was additionally regressed with baseline health-risk behaviours (smoking, binge drinking, and physical activity) at baseline (Model 2). The analyses were conducted with the TPHREG procedure in the SAS 9.1 statistical program package. All analyses were carried out separately for employees of =<50 years of age and >50 years of age and a test for age interaction was performed.

**Table 2:** Hazard ratios (HR) and their 95 percent confidence intervals (95% CIs) related to increased incidence of drug imbursement due to diabetes for initially healthy male employees =<50 years and >50 years of age during the 18-year follow-up according to the level of sense of coherence (SOC)

	***N (no of diabetes cases)***	Model 1*	**Model 2†**
		**HR (95% CI)**	**HR (95% CI)**
**Initial age =<50 years**		*p *for trend = 0.007	*p *for trend = 0.017
SOC			
Strong	1710 (81)	1.00	1.00
Medium	1799 (77)	0.95 (0.70–1.30)	0.94 (0.69–1.29)
Weak	1549 (96)	1.49 (1.08–2.07)	1.46 (1.05–2.03)
			
**Initial age >50 years**		*p *for trend = 0.961	*p *for trend = 0.958
SOC			
Strong	286 (19)	1.00	1.00
Medium	296 (24)	1.04 (0.58–1.86)	1.05 (0.56–1.94)
Weak	187 (16)	0.89 (0.42–1.85)	0.95 (0.44–2.02)

* Adjusted for age, education, marital status, psychological distress and self-rated health at baseline.

† Additionally adjusted for smoking status, binge drinking and physical activity at baseline.

## Results

A total of 313 (5.7%) participants were entitled to drug imbursement due to diabetes during the average follow-up of 17 years and 1 month (range 0.8–18 years). Of the participants, 558 (9.6%) died during the follow-up. For the participants who were entitled to drug imbursement due to diabetes during the follow-up, the mean before the entitlement was 13 years, with a range of 0.8–18.8 years. The distributions by baseline education, marital status, psychological distress, self-rated health and health-risk behaviours are presented in Table 1.

As can be seen from Table 1, in the regression models all socio-demographic characteristics predicted diabetes after the effect of age was controlled for. Low educational attainment and not being married independently increased the risk of becoming entitled to drug imbursement due to diabetes during the subsequent 18 years. In addition, being a smoker, binge drinking, and having a low level of physical activity were risk factors for subsequent diabetes medication.

After adjustment for age, educational attainment, marital status, psychological distress, self-rated health and health risk behaviours, a weak SOC was significantly associated with an increased risk of drug imbursement due to diabetes (HR = 1.36, 95% CI: 1.01–1.84; *p *for trend = 0.048) (data not shown). Table 2 presents the association between SOC and subsequent diabetes medication by age group. After adjustment for educational attainment, marital status, psychological distress and self-rated health, a weak SOC was significantly associated with an increased risk of drug imbursement due to diabetes in the employees who were =<50 years of age at baseline (HR = 1.46, 95% CI: 1.05–2.03). The similar pattern in results prevailed even after the models were further adjusted for health-risk behaviours. In contrast, a weak SOC did not predict drug imbursement due to diabetes among employees who were >50 years of age at baseline.

**Figure 2:**
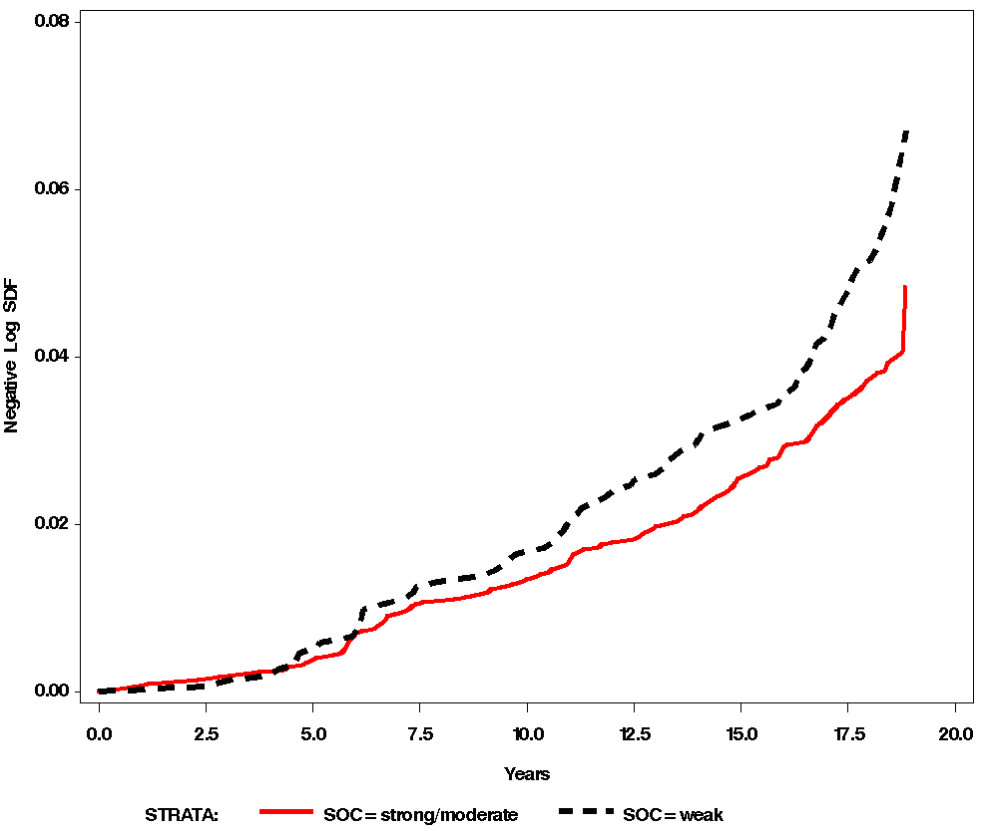
The predictive impact of sense of coherence (SOC) (weak vs. medium/strong SOC) on drug imbursement due to diabetes during follow-up of 18 years among initially healthy male employees =<50 years of age at baseline.

Figure 2 displays the survival curves representing two levels of SOC in the younger age group. It shows that as the level of SOC decreases, the risk of drug imbursement due to diabetes increases during the years of follow-up (log-rank test chi-square = 6.36, *p *= 0.01), the participants with a weak SOC having the shortest time before the entitlement to diabetes drugs. The men who had medium or strong level of SOC had a very similar risk of diabetes incidence during the follow-up and therefore these groups were combined in the Figure 2.

## Discussion

Although earlier research has shown that a weak SOC can predict general morbidity and mortality [11,13,19,32], only little is known whether it can predict specific severe chronic diseases [12]. As far as we know, there are no previous prospective studies of the relationship between SOC and diabetes. The results of this large scale longitudinal study among Finnish male employees showed that in a 18-year follow-up, initial weak SOC was associated with almost a 50% higher risk of diabetes in employees aged =<50 years at the start of the study. This result could not be explained by a variety of controlled baseline socio-demographic, psychosocial and health-risk behaviour variables. Therefore these results suggest that there can be a direct relationship between a weak SOC and physiological consequences affecting health. The result is in accordance with two earlier cross-sectional studies indicating a relationship between SOC and diabetes [17,18].

Moreover, our findings are in line with recent prospective evidence showing a link between a weak SOC and an increased risk of disability pension among employees =<50 years of age in entry of study [19], although it should be noted that most of the principal causes for disability pensions are not directly related to diabetes.

We did not find a significant association between SOC and diabetes in the older age group (>50 year-olds). With increasing age the sensitivity of SOC towards diabetes might decrease since the risk of many different kinds of diseases is concomitantly increasing in older people which again also might influence the SOC scores. Furthermore, it is possible that poor health and other bio-medical risk factors related to diabetes are more likely to explain the onset of diabetes in older industrial employees. In line with the results by Suominen *et al*. [19], our findings propose that the health-damaging mechanisms related to weak SOC, leading to a higher risk of diabetes, already take place at rather early age.

There are at least three processes that may explain the link between SOC and health [33]. First, common genetic or physiological processes can determine both psychological attributes and disease. Second, individual differences (personality traits or attributes), such as SOC, can influence health promoting (e.g., regular exercise) or damaging (e.g., unhealthy diet) behaviours. Third, individual characteristics can influence the effective implementation of health-related coping behaviour and have an adverse effect on mental health.

Recent research supports Antonovsky's theory that SOC moderates (buffers) the health impacts of adverse life events [34]. A weak SOC might increase experiences of overwhelming and negative stress through autonomous neural pathways, by neuroendocrinological or neuroimmmunological mechanisms [35], and this could be reflected in a higher risk of diabetes. A weak SOC can be interpreted as a lower ability to cope with stressors [7,17]. Exposure to long-term stress affects the entire neuroendocrine system, activating the hypothalamic-pituitary-adrenal (HPA) axis and the central sympathetic nervous system [36]. Increased cortisol levels following activation of the HPA axis could play a role in the development of decreased glucose tolerance. Cortisol has been shown to induce insulin resistance by increasing hepatic glucose production, suppressing glucose usage, and inhibiting insulin secretion [37].

SOC is strongly linked with aspects of negative emotionality [10,38,39]. Negative emotionality has been associated with higher body mass index and weight gain [40] and hostility with the so-called 'metabolic syndrome'[41], which are all risk factors for Type-2 diabetes. Additionally, people with a weak SOC can perceive their environment as nonsupportive. A weak SOC has been associated with low social support [42], which has in turn been found to increase the risk of poor health [43].

To sum up, a potential combination of stress inducing reactive tendency, inadequate coping systems and unhealthy lifestyle choices characterizing those with a weak SOC may help to explain the association between SOC and diabetes identified in this study.

### Strengths and limitations

The cohort size of this study, our capacity to adjust for several traditional risk factors for diabetes, together with reliable prospective ascertainment of entitlement to drug imbursement due to diabetes from national registers provided a unique opportunity to test and confirm the hypothesis that a weak SOC is associated with an increased risk of diabetes. Non-response occurred randomly enough to limit the potential for selection bias. In addition, the observed effect size in employees =<50 years of age was relatively large, a 46% increase in the incidence of diabetes (after adjustment for several traditional risk factors). Further advantages include an exceptionally long the follow-up period. Earlier prospective studies on SOC and objective health outcomes have been based on less than 10 years follow-up periods. The use of long enough follow-up is important as the influence of SOC on severe health outcomes, such as diabetes, is slow to manifest itself.

However, our results should be interpreted in light of some limitations. First, even if a long follow-up can generally be considered as strength, on the other hand individuals can develop other health problems or poor health behaviours during this lengthy time period, and these problems and/or behaviours may have an impact on the eventual development of diabetes.

Second, some patients with Type-2 diabetes do not use medication but try to control their disease with a help of proper diet and exercise. These individuals would not have been identified in this study design. However, due to well developed health screening and occupational health care system in Finland, we can be fairly certain that most cases entitled to reimbursement of diabetes medication were detected in our study. It is possible, nevertheless, that employees who died during the follow-up, especially some of those employees who had a cardiovascular diagnosis (*N *= 185), may have had an undetected impaired glucose tolerance which may have contributed as a risk factor for death. If this is the case, the effect of weak SOC on diabetes incidence found in this study may be an underestimate.

Third, the Drug Imbursement Register was used as a means of determining the presence of diabetes and other chronic diseases at baseline. However, there are chronic diseases for which medications are not common or for which medications were developed only in recent years. In spite of this, the Drug Imbursement Register can be seen as a fairly reliable and objective data to use in determining a "healthy" status.

Fourth, in this study our outcome was disease even though theoretically SOC is promotive of health rather than illness.

Fifth, the SOC theory is not very specific about diagnoses, whereas our study was specific about the studied outcome. Individual characteristics such as SOC are typically generalised resistance resources, that is, their effects on health are non-specific [44]. When encountering stressors and adverse life events, individuals can react differently and with different health outcomes because of their resources and other characteristics (e.g., work characteristics) [44,45]. However, in a medical study it is very difficult to include the incidences of diseases that have very different biomedical pathogenesis, such as for example type 2 diabetes and depression, in the same outcome variable. In our opinion, it is important to widen the scope of SOC theory towards various biomedical outcomes, such as coronary heart disease, diabetes or other conditions, and to further specify the psychomedical, psychosocial and psychobehavioral mechanisms through which SOC may affect various health and ill health outcomes, even though this is a dilemma in relation to traditional SOC research since Antonovsky's theory is not diagnosis-specific.

Sixth, the most serious epidemiological shortcoming of our study was that our questionnaire did not include questions about dietary habits, body mass index (BMI) and weight gain and therefore we were unable to examine their mediating role. In previous studies SOC has been associated with unhealthy lifestyles, such as unhealthy food choices [16]. It is possible that the lack of weight-related variables in the analyses have affected the results. Further research needs thus to be conducted to test whether dietary habits, BMI or weight gain could be responsible for the association between SOC and diabetes.

Seventh, our physical activity measure was not optimal for this kind of study since the response options were not specific enough to separate sufficiently people with different frequency of weekly exercise. This shortcoming becomes even more significant since the study did not include data on BMI. Those participants exercising the most frequently are probably the most unlikely to be overweight.

However, regular physical exercise at least once a week has been associated with a reduced risk of type 2 diabetes in initially healthy 35–60-year-old men [28]. Moreover, another study [46] found that for those older adults who were physically active once a week, the risk of all-cause mortality was 40% lower than for those who were physically inactive. For those who were physically active more frequently, the reduction in all-cause mortality risk was about the same as for those who were physically active once a week.

Finally, assessing health behaviours only at baseline can be a limitation as these behaviours may change over 18 years. Finally, it can be assumed that individuals' health may influence their SOC, i.e., the causality between SOC and health may also operate in the other way around. Health represents one of the sources responsible for the maintenance of the level of SOC [7]. However, in earlier research predictive relationships from health to SOC has not been found [11].

Further studies in other countries and in other sectors are needed to confirm and develop our findings as well as determine their generalisability. More research is especially needed to examine the biopsychosocial mechanisms behind the association between SOC and diabetes, and investigate the associations of other individual difference variables with diabetes.

## Conclusion

The figures from the WHO show that the number of people with diabetes will at leas more than double by 2030. The human and economic costs of this trend are enormous [2]. Given that Type-2 diabetes is partly preventable, it is important to identify also psychological factors that can contribute to health [6]. Our results provide new evidence that a weak SOC predicts diabetes in male employees who were =<50 years or age in entry into the study. Besides focusing on well-known behavioural risk factors for diabetes, strengthening SOC can also play a role in attempts to tackle increasing rates of diabetes.

Individual characteristics such as SOC are typically generalised resistance resources, that is, their effects on health are non-specific [44]. As a consequence, interventions aimed at increasing resources can have a positive effect on a wide range of outcomes. In other words, strengthening SOC can reduce the risk of also other health problems than diabetes, at least of those with similar biomedical pathogenesis. Individual level interventions [47] can be used to strengthen SOC. Furthermore, in employee populations, workplace interventions targeting such factors as organisational climate and leadership relations could be useful as changes in these factors have been associated with changes in SOC [48]. According to the SOC theory, high SOC is more stable than weak SOC and it thus is possible that work characteristics are more likely to cause modificiations in SOC in persons who score low on the SOC-scale than in persons who score high on the SOC-scale [49]. Therefore employees with low SOC could especially benefit of positive changes in their work environment.

## Abbreviations

ATC = Anatomic Therapeutic Chemical; CI = confidence interval; HR = hazard ratio; SD = standard deviation; SOC = sense of coherence; WHO = World Health Organization.

## Competing interests

The author(s) declare that they have no competing interests.

## Authors' contributions

AMK and AV together designed the study and planned the data analyses. AMK directed the implementation of the study and was the principal author of the paper. AV carried out the data analyses and contributed to interpreting the results and writing the paper. SAW, TH and ST-T contributed to interpretation of the results and manuscript writing. AK was involved in the data collection, constructed many of the measures and helped to prepare Materials and Methods section. All authors read and approved the final manuscript.

## Pre-publication history

The pre-publication history for this paper can be accessed here:


